# The oncolytic effect *in vivo* of reovirus on tumour cells that have survived reovirus cell killing *in vitro*

**DOI:** 10.1038/sj.bjc.6603363

**Published:** 2006-10-03

**Authors:** T Alain, M Kim, R N Johnston, S Urbanski, A E Kossakowska, P A Forsyth, P W K Lee

**Affiliations:** 1 Department of Medical Sciences, University of Calgary, Calgary, Alberta, Canada; 2 Department of Pathology, University of Calgary, Calgary, Alberta, Canada; 3 Department of Microbiology and Immunology, Dalhousie University, 7/F Sir Charles Tupper Building, 5850 College Street, Halifax, Nova Scotia, Canada B3H 1X5

**Keywords:** reovirus oncolysis, persistent infection, cured cells, tumour regression

## Abstract

The use of oncolytic viruses has received considerable attention in recent years and many viruses have proved to be effective against a variety of cancer models and a few are currently being used in clinical trials. However, the possible emergence and outcome of virus-resistant tumour cells has not been addressed. We previously reported the effective use of reovirus against lymphoid malignancies, including the Burkitt's lymphoma cell line Raji. Here we isolated *in vitro* persistently infected (PI) Raji cells, and cells ‘cured’ of persistent reovirus infection (‘cured’ cells). Both PI and cured Raji cells resisted reovirus infection and cell killing *in vitro*. *In vivo*, the PI cells were non-tumorigenic in SCID mice, but cured cells regained the parental cells' ability to form tumours. Tumour xenografts from the cured cells, however, were highly susceptible to reovirus oncolysis *in vivo*. This susceptibility was due to the proteolytic environment within tumours that facilitates reovirus infection and cell killing. Our results show that persistent infection by reovirus impedes tumour development and that although PI cells cleared of reovirus are tumorigenic, they are killed upon rechallenge with reovirus. Both the PI and cured states are therefore not likely to be significant barriers to reovirus oncolytic therapy.

Reovirus is the prototype of the Reoviridae family of double-stranded RNA viruses that is found ubiquitously in the environment. Although most humans have been infected with this virus, there is little or no manifestation of clinical symptoms (Tyler and Fields, 1996). The virus is not linked to any known human diseases and is therefore considered to be benign. More recently however, reovirus has been shown to be an effective oncolytic agent against a variety of cancers (Wilcox *et al*, 2001; Hirasawa *et al*, 2002; Norman *et al*, 2002; Etoh *et al*, 2003; Kilani *et al*, 2003) including lymphomas (Alain *et al*, 2002). These studies led to clinical trials of reovirus as a cancer therapeutic (Morris *et al*, 2002).

While the use of viruses as oncolytic agents has shown considerable promise in preclinical studies (Kirn, 2002; Bell *et al*, 2003), the possibility of generating or selecting for cancer cells that have become resistant to reovirus has not been evaluated. It is possible that a subpopulation of cancer cells may survive the initial viral assault and become persistently infected (PI) with the virus. Although it is not known if such residual cancer cells actually exist *in vivo*, the fact that cells PI with reovirus can be established *in vitro* (Taber *et al*, 1976; Ahmed *et al*, 1981; Ahmed and Fields, 1982; Danis *et al*, 1993; Dermody *et al*, 1993, 1995; Dermody, 1998) suggests that this could be a potential problem *in vivo*. It is therefore important to evaluate the tumorigenicity of such PI cells relative to that of the parental cells to see if this could represent a mechanism for proliferating tumour cells to escape initial viral oncolysis.

Another more important concern pertains to the potential generation, from the PI cells, of cells ‘cured’ of viral infection. In the case of reovirus, cured cells can be generated from PI murine L cells simply by incubation with anti-reovirus antibodies. Importantly, these cells then lack permissiveness to wild-type reovirus *in vitro* (Ahmed *et al*, 1981; Dermody *et al*, 1993; Baer and Dermody, 1997; Wetzel *et al*, 1997; Baer *et al*, 1999). Whereas the presence of viruses in PI cells may keep tumour cells in check, cured cells without such a deterrent could potentially develop into tumours. If cured tumours are now resistant to the original wild-type virus, this may pose another potential therapeutic challenge.

In this study, we addressed these issues using the Burkitt's lymphoma cell line Raji, which we have found to be highly susceptible to reovirus oncolysis (Alain *et al*, 2002). Despite this exquisite susceptibility *in vitro*, we isolated a resistant population that was PI, and generated cured cells from this PI population using anti-reovirus antibodies. We compared the PI and cured cells to the parental cells in terms of tumorigenicity and susceptibility to reovirus oncolysis *in vitro* and *in vivo*. We found that PI Raji cells were nontumorigenic, but cured cells formed large tumours *in vivo*. Raji cured cells were resistant to reovirus infection *in vitro*, but were highly susceptible to reovirus oncolysis *in vivo*. Subsequent experiments demonstrated that the presence of proteases within the microenvironment of Raji cured tumours enhanced the processing and hence the oncolytic activity of reovirus *in vivo*. In view of the lack of tumorigenicity of the PI cells and the susceptibility of cured cells to reovirus *in vivo*, we conclude that these conditions should not represent a significant barrier for reovirus oncolytic therapy.

## MATERIALS AND METHODS

### Cells and reovirus

The human Burkitt's lymphoma cell line Raji, obtained from American Type Culture Collection (ATCC), was maintained in RPMI containing 10% foetal bovine serum (FBS), 1% L-glutamine (Gibco/BRL, Burlington, ON, Canada) and antibiotics (Sigma-Aldrich, St Louis, MO, USA). Cells were kept at 37°C in a humidified 5% CO_2_ incubator. For the growth curve assay, Trypan Blue exclusion test was performed every 24 h. The cells were stained with 0.25% dye and viable cells in three independent wells were counted using a haemocytometer.

The PI cells were established after infection of the parental Raji cells with reovirus type 3 Dearing and isolating the surviving populations. The surviving cells were subsequently re-infected three times a week for 3 weeks at 20 plaque-forming units (PFU) per cell to eliminate all cells susceptible to lysis. The cells then underwent two periods of crisis in which the majority of cells died, but resistant cells eventually emerged and remained PI (Raji PI). Raji cured cells were generated by culturing the PI cells in the presence of 1% (v/v) filter sterilised polyclonal rabbit anti-reovirus serotype 3 antiserum. Cells were passaged in the presence of antiserum every second day for a period of 2 weeks. The cell population obtained following this treatment contained no detectable viral particles.

The Dearing strain of reovirus serotype 3 was propagated in L929 cells grown in suspension in Joklik's modified Eagle's medium (JMEM) containing 5% FBS. The virus was purified according to the protocol of Smith *et al* (1969), with the exception that *β*-mercaptoethanol was omitted from the extraction buffer.

Infectious subviral particles (ISVPs) were obtained by digesting virions (1 × 10^11^ PFU ml^−1^) with *N*-*α*-*ρ*-tosyl-ι-lysine chloromethyl ketone (TLCK)-treated *α*-chymotrypsin (200 *μ*g ml^−1^) (Sigma-Aldrich) for 30 min at 37°C. Digestion was stopped by the addition of ethanolic phenymethysulphonyl fluoride (5 mM) (Sigma-Aldrich) at 4°C, and the ISVPs generated were used immediately for cell infection.

### Polymerase chain reaction and immunofluorescence

Cells were harvested and RNA was extracted using the RNeasy protocol (Qiagen Inc., Missassauga, ON, Canada). Equal amounts of total cellular RNA from each sample were then subjected to RT–PCR as described previously (Strong *et al*, 1998). S1 cDNA primers: 5′-AATTCGAATTAGGTGACACTATAG-CTATTGGTCGGATG-3′ and 5′-CCCTTTTGACAGTGATGCTCCGTTATCACTCG-3′. S2 cDNA primers: 5′-TTCGCTGGTCAGTTATGGCTC-3′ and 5′-TACCAGCTCGGAGTAGATGTG-3′.

For immunofluorescent studies, cells were spread and fixed on slides using Cytology Fixative (Surgipath, Richmond, IL, USA). After rehydration by sequential washes in 75, 50 and 25% ethanol followed by four washes with phosphate-buffered saline (PBS), the fixed and rehydrated cells were treated with 10% goat serum in PBS and then exposed to the primary antibody (rabbit polyclonal anti-reovirus type 3 serum diluted 1/5000 in PBS) overnight at 4°C. Following three washes with PBS, the cells were exposed to the secondary antibody (fluorescein isothiocyanate (FITC)-conjugated goat anti-rabbit IgG diluted 1/100 in PBS) for 1 h at room temperature. The slides were then washed three times with PBS, mounted with DAPI mounting medium (Vector Laboratories, Burlingame, CA, USA), and the cells were viewed and photographed with a Zeiss microscope (magnification × 400).

### Ras activation assays and Western blot analysis

Ras-GTP levels were measured using a Ras activation assay kit (Upstate Biotechnology, Lake Placid, NY, USA). Subconfluent cells in 15-cm dishes were washed two times in ice-cold TBS and lysed by scraping in 500 *μ*l of Mg^2+^ lysis buffer (MLB, Upstate Biotechnology). Six hundred microlitres of precleared lysate was then incubated with 30 *μ*l of GST–Raf Ras-binding domain bound to glutathione agarose for 45 min at 4°C. Beads were then washed three times in MLB and boiled in 40 *μ*l of 2 × protein sample buffer with 2 *μ*l of 1 M DTT. Twenty microlitres of pull-down sample and 40 *μ*l of total lysate were then subjected to 10% SDS–PAGE and transferred to nitrocellulose membrane. Blots were probed overnight with 1000-fold diluted RAS10 mAb (Upstate Biotechnology) and then washed three times for 5 min in TBS/0.1% Tween-20, followed by incubation in goat anti-mouse-HRP Ab (1 : 2500, Santa Cruz Biotechnology, Santa Cruz, CA, USA). After three more washes, blots were processed and signals were visualised using enhanced chemiluminescence (Amersham Biosciences, Little Chalfont Buckinghamshire, England).

For detection of activated extracellular signal-regulated kinase (ERK), cells in complete medium were washed in ice-cold TBS and lysed in standard radioimmunoprecipitation assay buffer with phosphatase inhibitors (TBS, 0.1% SDS, 0.5% sodium deoxycholate, 1% Triton X-100, 10 mM sodium pyrophosphate, 25 mM *β*-glycerophosphate, 1 mM, sodium orthovanadate, 25 mM sodium fluoride). Forty microlitres of precleared lysate were then analysed by Western blot using the anti-phospho-ERK1/2 and anti-total ERK1/2 Abs obtained from Cell Signaling Technology (Beverly, MA, USA).

### Soft agar assay

A total of 1 × 10^5^ cells of parental, PI and cured cells were mixed (1 : 1) in 2 × RPMI containing 10% FBS and 1.2% low-melting temperature agarose (SeaPlaque, Rockland, ME, USA) and allowed to grow for 4 weeks. Colonies were then fixed in ethanol/acetic acid overnight and stained with Coomassie brilliant blue. Photomicrographs of the wells were taken on a Kodak DC290 ZOOM digital camera.

### Reovirus infection of the Raji cell lines *in vitro*

One million cells of each cell line were dispensed into 24-well plates and infected with reovirus or ISVPs at an MOI of 20 PFU cell^−1^. For experiments involving the use of proteases, infection with reovirus was carried out in the presence of 10 *μ*g ml^−1^ TLCK-treated chymotrypsin (replaced every 24 h for up to 96 h). For E64 protease inhibitor (Sigma) treatment *in vitro*, cells were exposed to 50 *μ*M of E64 in PBS 1 h before reovirus infection. Virus was allowed to bind for 45 min at 4°C, after which E64 was again added to the cells to a final concentration of 50 *μ*M.

WST-1 (4-[3-(4-iodophenyl)-2-(4-nitrophenyl)-2*H*-5-tetrazolio]-1,3-benzene disulphonate) assays (Roche, Diagnostics, Laval, Qc, Canada) were used to measure cell viability. Cells were infected as above and a tetrazolium salt WST-1 was added to the cultures for 6 h before the 96 h time point postinfection. The cleavage of WST-1 to formazan by metabolically active cells was quantified by scanning the plates at 450 nm reference wavelength in a microtitre plate reader. Medium without WST-1 was used as background control. The experiments were performed in triplicate.

For metabolic labelling, [^35^S]methionine was added to the culture medium at designated times for a period of 6 h. Cells were harvested and lysed in lysis buffer (PBS) containing 1% Triton X-100, 0.5% sodium deoxycholate and 1 mM EDTA). Lysates were cleared of debris by centrifugation and supernatants were stored at −70°C until use. Polyclonal rabbit anti-reovirus serotype 3 serum was used for immunoprecipitation of [^35^S]methionine-labelled reovirus proteins from cell lysates as described previously (Lee *et al*, 1981). Immunoprecipitated proteins were subjected to SDS–PAGE (Laemmli, 1970) followed by autoradiography.

### Progeny virus production

Three million cells grown in six-well plates were infected with reovirus at an MOI of 20. At 96 h postinfection, the plates were frozen and stored at −70°C until use. To assay for progeny virus production, the plates were subjected to three rounds of freeze–thaw, and the supernatants were used for plaque titration on L929 cells. All titration experiments were repeated in triplicate.

### SCID mice studies

Six- to 8-week-old Fox-Chase SCID mice were obtained from the Jackson Laboratory (Bar Harbor, ME, USA). The animals were maintained under specific pathogen-free conditions and according to a protocol approved by the University of Calgary Animal Care Committee. As a xenograft model, 1.0 × 10^7^ Raji or Raji-derived cells (PI or Cured) were injected subcutaneously in the hind flank of the mice. Once palpable tumours were established (day 0), 1.0 × 10^7^ PFU of live reovirus in PBS was administered intratumorally (experimental group), or PBS alone (control group). Two-dimensional tumour measurements were performed with calipers every other day for 25 days or until the animals showed severe morbidity due to excess tumour burden or complications due to viral infection.

*In vivo* experiments involving the use of the protease inhibitor E64 were similarly carried out, with the exception that 1 mg of E64 in PBS was injected IP into the mouse and subsequently every second day, until day 8 for the E64 alone and the E64+reovirus group. This concentration was chosen from a previous report showing the inactivation of proteases by E64 *in vivo* without affecting the tumour growth of a Burkitt lymphoma tumour (Sebti *et al*, 1991). For the E64+reovirus group, E64 injection was performed 1 h before the single reovirus injection. For ISVP treatment, ISVPs were prepared as described above and 1.0 × 10^7^ PFUs were administered once intratumorally.

### Histology and immunohistochemistry

Tumours (or remaining masses) taken from animals on day 20 post-intratumoral reovirus (or saline) injection were fixed in 10% neutral-buffered formalin and embedded in paraffin for histological analysis. Sections were then immersed in xylene, followed by rehydration in decreasing concentrations of ethanol. For histopathological examination, the sections were stained with haematoxylin and eosin (H&E). For immunohistochemistry, endogenous peroxidase was inactivated in 3% hydrogen peroxide in methanol for 15 min. Sections were then incubated with a primary rabbit anti-reovirus polyclonal antibody (1/1000 in PBS with 10% goat serum and 0.1% Triton X-100) that was partially purified by ammonium sulphate precipitation. Slides were washed in PBS and then subjected to avidin–biotin–horseradish peroxidase staining as recommended by the manufacturer (Vector, Burlingame, CA, USA) and counter-stained in haematoxylin.

### Statistical analysis

Statistical Analysis Software GraphPad Prism (version 4, GraphPad Software Inc., San Diego, CA, USA) was used for statistical analyses. Wilcoxon's signed-rank tests were used to compare the distributions of tumour sizes. All reported *P*-values were two-sided and were considered to be statistically significant at <0.05.

## RESULTS

### Growth of PI, cured and parental Raji cells *in vitro*

To generate PI cells, parental Raji cells were subjected to multiple rounds of infection by reovirus type 3 Dearing. Most cells were killed by the virus, but a very small number of cells survived and were propagated for the next several months. After undergoing two crisis periods, a resistant population was established that was PI and that continuously shed infectious viral particles into the culture medium. These cells (designated Raji PI) could subsequently be cured of reovirus infection using anti-reovirus antibodies. Cured cells (designated Raji cured) contained no detectable viral proteins or RNA (see below). Evaluation of the cluster of differentiation antigens present on PI and cured cells verified their common origin from Raji cells (data not shown).

**Figure 1:**
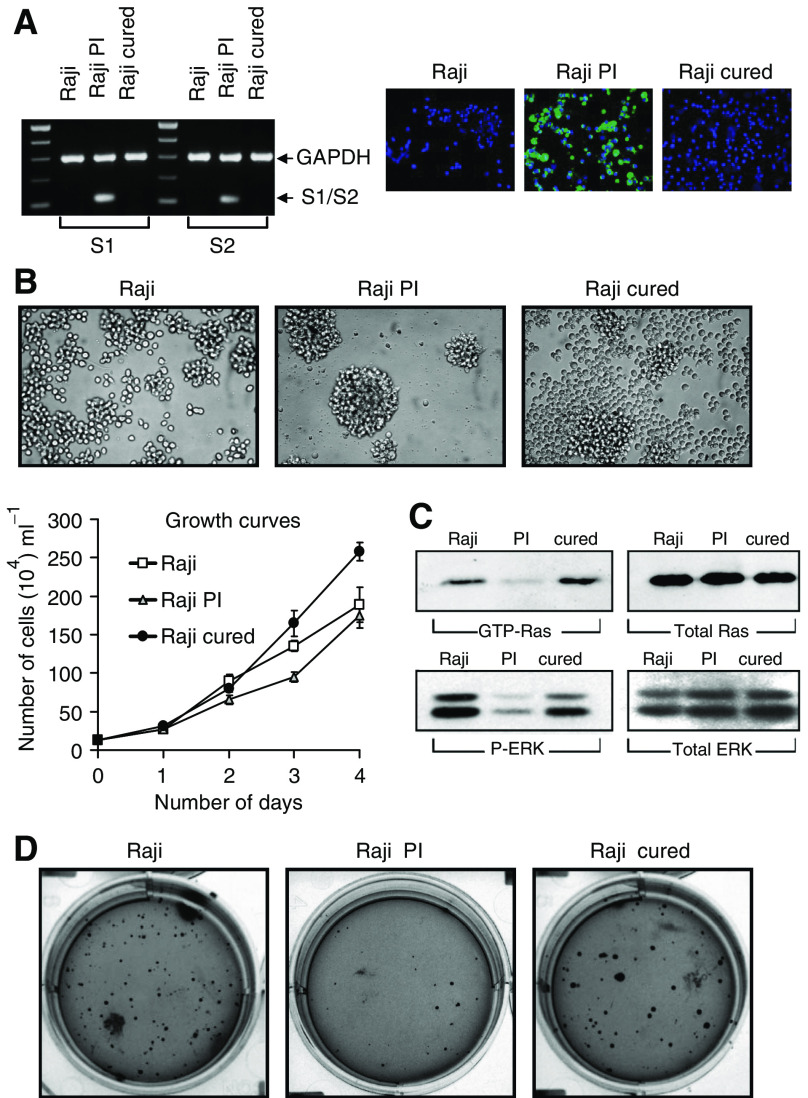
Growth of the Raji parental, PI and cured cells. (**A**) The left panel shows polymerase chain reaction of reovirus S1 and S2 mRNA transcript in Raji parental, PI and cured cells. Equal amounts of RNA from each sample were subjected to RT–PCR, followed by selective amplification of reovirus S1 or S2 cDNA and GAPDH. The right panel shows immunofluorescence of reovirus proteins expressed only in PI cells. Cells grown under normal conditions were fixed, processed and reacted with rabbit anti-reovirus type 3 antibody, followed by FITC-conjugated goat anti-rabbit IgG and mounted with DAPI-stained mounting medium. The magnification for all panels was × 400. (**B**) Photomicrographs of the Raji parental, PI and cured cells in culture and growth curves assessed by staining the cells with 0.25% Trypan Blue. Viable (unstained) cells from three independent wells were counted using a haemocytometer. (**C**) Ras and ERK activity in Raji, PI and cured cells. (**D**) Growth of Raji parental, PI and cured cells in soft agar. A total of 1 × 10^5^ cells were mixed (1 : 1) in 2 × RPMI containing 10% FBS and 1.2% low-melting temperature agarose (SeaPlaque) and allowed to grow for 4 weeks. Colonies were then fixed, stained with Coomassie brilliant blue and photomicrographed.

We first characterised and compared the Raji PI and Raji cured cells to the parental Raji cells. RT–PCR and immunofluorescence confirmed the presence of reovirus only in the PI cells (Figure 1A); no viral transcripts or proteins were detectable in Raji cured cells. Persistently infected cells also tended to form clusters in culture and grew at a slightly slower rate while the proliferation rate of the cured cells surpassed that of the parental cells (Figure 1B). Examination of activated Ras-GTP and phosphorylated ERK1/2 levels revealed that both were significantly reduced in PI cells, but were restored in the cured cells to those seen for the parental cells (Figure 1C). Moreover, cured and parental Raji cells readily formed colonies in soft agar, whereas PI cells did not (Figure 1D); this suggests that the cured cells, like the parental cells, are likely tumorigenic whereas the PI cells are likely nontumorigenic.

### Parental and cured cells, but not PI cells, develop large tumours *in vivo*

**Figure 2:**
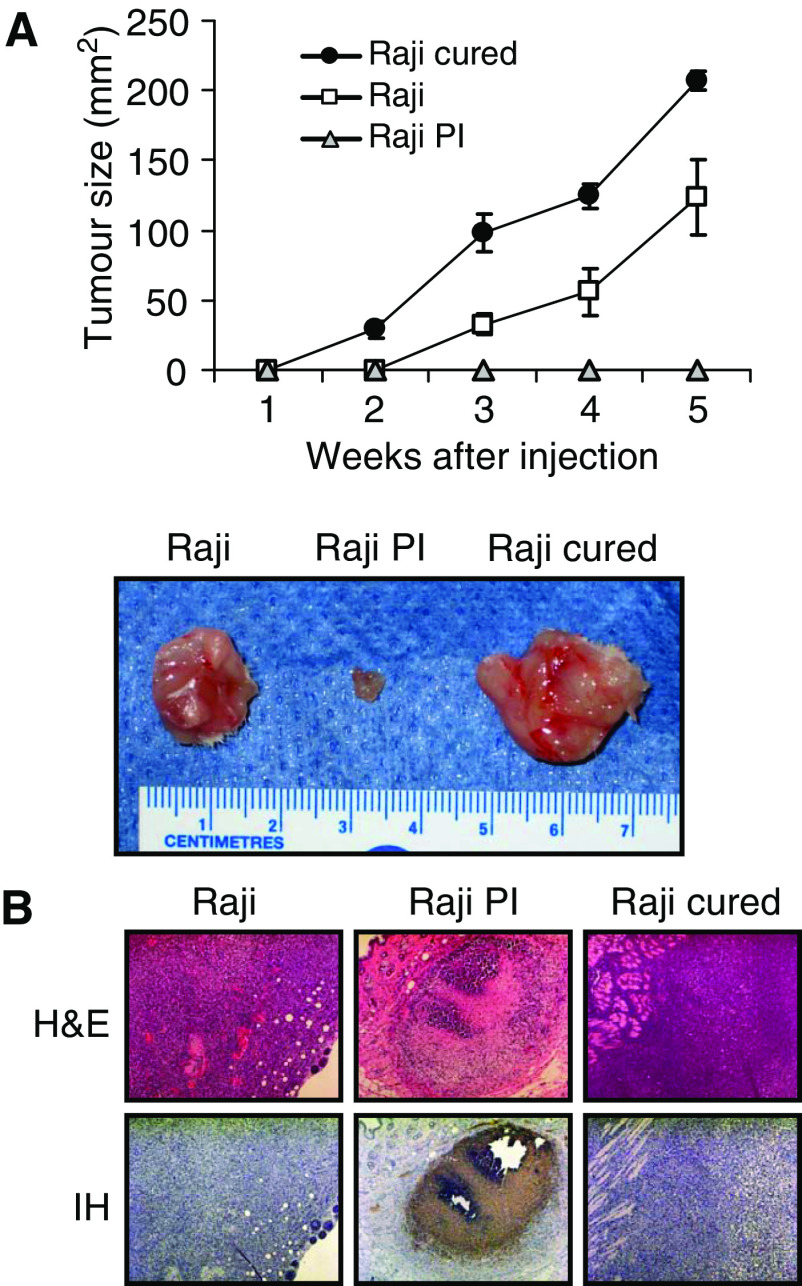
Tumorigenicity of Raji parental, PI and cured cells in SCID mice. (**A**) SCID mice were subcutaneously implanted with 1 × 10^7^ cells of Raji parental, PI or cured cells. Tumour growth was followed for a period of 5 weeks and two-dimensional measurements were taken weekly with a caliper. Resected tumours were photographed. (**B**) Haematoxylin and eosin staining and IH of reovirus antigens in tumour tissue taken from Raji parental, PI and cured tumours. Haematoxylin- and eosin-stained section (original magnification × 200) show large tumours 30 days postinjection in the Raji and cured group. Tumours in the PI group were not apparent on palpation and only small foci of tumour were found on histological examination. Immunohistochemical of tumour sections (original magnification × 200) shows that only PI cells stain positively for reovirus proteins (brown), although most of the tumour is necrotic.

Despite numerous studies on reovirus PI cells, the tumorigenicity of PI and cured cells has never been reported. Accordingly, the cells were introduced subcutaneously into SCID mice, which had previously been shown to support the growth of Raji tumours. The results (Figure 2A) show that, in agreement with the *in vitro* soft agar assay, both the parental and cured cells produced large tumours, whereas Raji PI cells failed to grow in these mice. Histological examination showed large and clearly proliferating tumours in the Raji parental and Raji cured groups. The PI group had small tumours that could not be palpated and were detectable only microscopically by immunohistochemistry using anti-reovirus antibodies (Figure 2B). It is interesting to note that the abilities of the three cell types to form tumours correlated with their Ras-GTP and ERK1/2 levels.

### Raji cured cells are resistant to reovirus oncolysis *in vitro* but are susceptible *in vivo*

**Figure 3:**
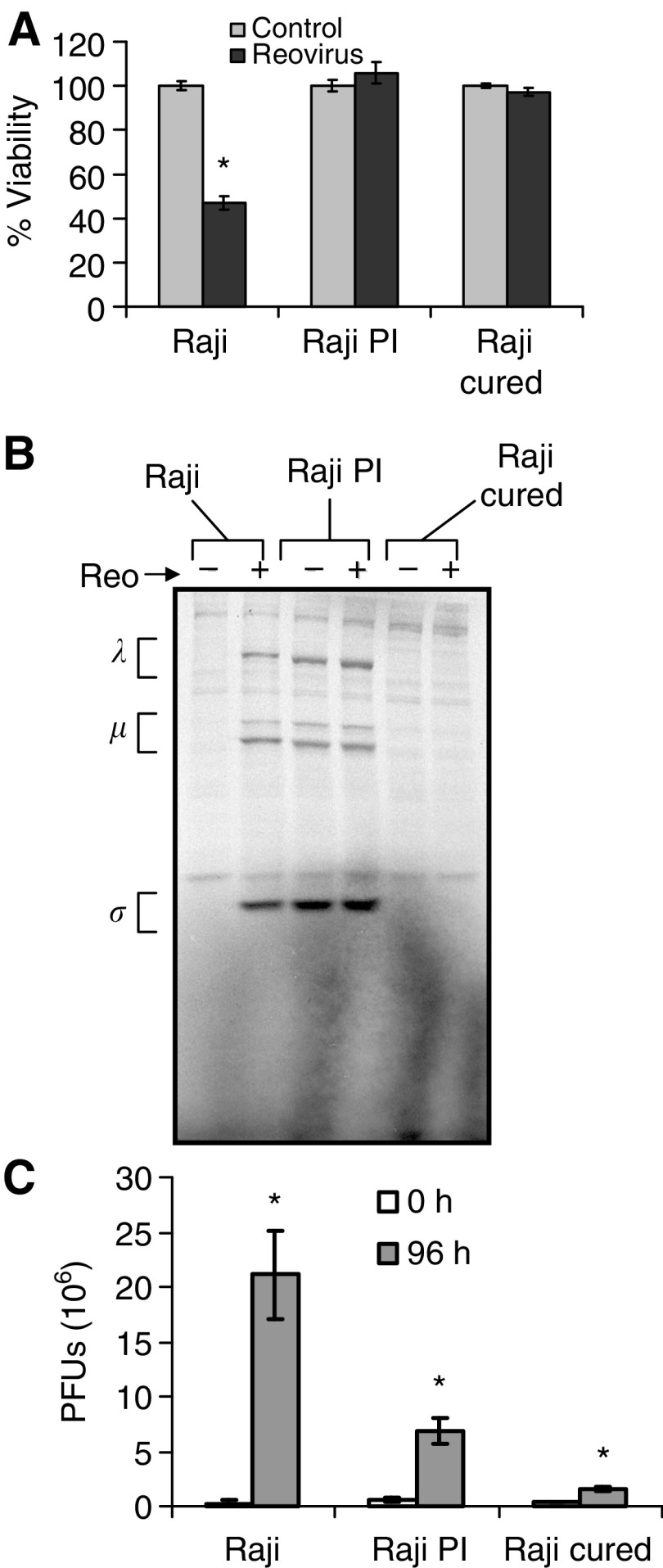
Effect of reovirus on Raji parental and cured cells *in vitro*. (**A**) Cell viability. Raji parental, PI and cured cells were exposed to reovirus at an MOI of 20 PFU cell^−1^, and viability at 96 h postinfection was assessed using WST-1 assay from three independent wells. Compared to controls, *P*-values for reovirus-treated groups are *P*<0.001, *P*=0.93 and *P*=0.222 for Raji, Raji PI and Raji cured, respectively. (**B**) Viral protein synthesis. Infected cells were metabolically labelled with [^35^S]methionine for 6 h at 42 h postinfection. Cell lysates were prepared, and reovirus proteins were immunoprecipitated with a polyclonal anti-reovirus antibody and analysed by SDS–PAGE. The three size classes of reovirus proteins (*λ*, *μ* and *σ*) are indicated on the left. (**C**) Virus progeny. At 0 and 96 h postinfection, cells were harvested and freeze–thawed three times, and the virus titre in the lysate was determined by plaque assay on L929 cells. Compared to input virus, *P*-values for progeny virus at 96 h postinfection are *P*<0.001, *P*=0.0015 and *P*<0.001. Error bars indicate s.e.m. from three separate wells and ^*^demonstrates statistical significance.

To determine the permissiveness of the Raji cured cells to reovirus infection *in vitro*, the cells were infected with reovirus at an MOI of 20 PFU cell^−1^ and assessed for cytopathic effects, viability, viral protein synthesis, and progeny virus production. As reported previously, Raji parental cells became granular and irregular-shaped, and manifested extensive cell clumping characteristic of cytopathic effects following reovirus infection. Cured and PI cells were however resistant to reovirus even at 96 h postinfection. Based on WST assay performed on the cells at this time point, viability was reduced in the parental cell line to approximately 45%, whereas no change in viability was detected in the cured or PI cells (Figure 3A).

The infected cells were also metabolically labelled with [^35^S]methionine, followed by immunoprecipitation with anti-reovirus antibody for the detection of viral protein synthesis (Figure 3B). The synthesis of the three viral protein groups (*λ*, *μ* and *σ*) was observed in the parental Raji cells, but not in the Raji cured cells. Resistance of the cured cells to reovirus infection was also evident from the lack of progeny virus production even at 96 h postinfection compared to the parental cells (Figure 3C). As expected, the PI cells supported endogenous viral protein synthesis, which did not appear to be enhanced by the addition of exogenous reovirus (Figure 3B). The continuous production of endogenous virus in PI cells was accountable for the moderate enhancement in virus yield with time (Figure 3C).

**Figure 4:**
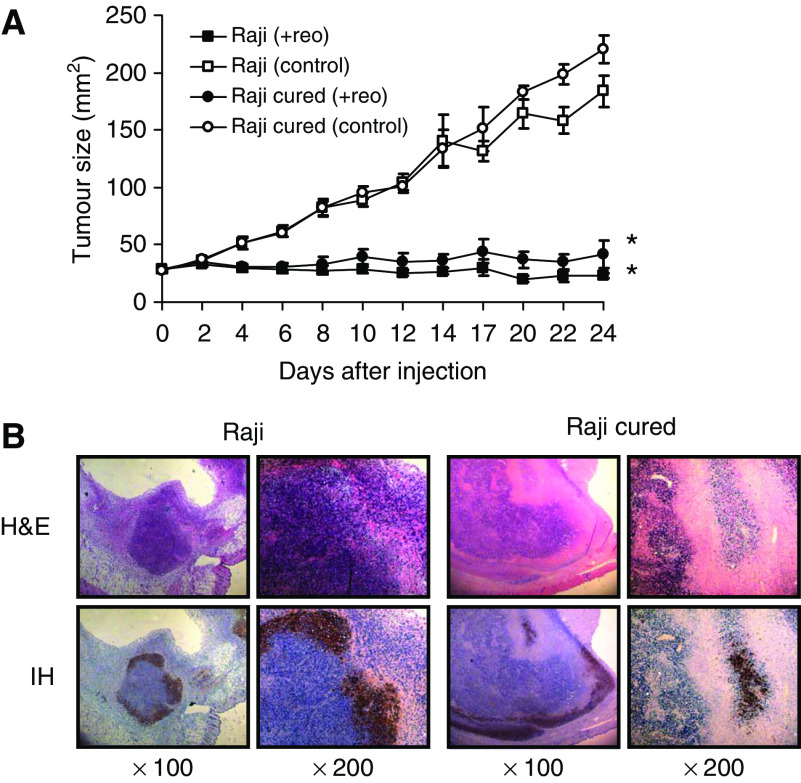
Effect of reovirus on parental and cured Raji tumours *in vivo*. (**A**) Intratumoral reovirus therapy of lymphoid tumours in SCID mice. SCID mice were subcutaneously implanted with 1 × 10^7^ cells of either Raji parental or cured cells. Following palpable tumour establishment, the tumours received (on day 0) a single intratumoral injection of 1 × 10^7^ PFUs of live reovirus (*n*=8) or saline control (*n*=7). Tumour growth was followed for a period of 25 days and measured two-dimensionally with a caliper. ^*^Differences are statistically significant with *P*<0.001 for Raji+reovirus and *P*=0.002 for Raji cured+reovirus compared to respective controls. (**B**) Haematoxylin and eosin staining and IH of reovirus antigens in Raji parental and cured tumours after intratumoral reovirus treatment. Haematoxylin- and eosin-stained section shows necrosis of tumour cells 24 days after live reovirus treatment. IH-stained section (original magnification × 100 (left) and × 200 (right)) of remaining tumour cells stains positively for reovirus proteins (brown). Histological examination of saline-treated (control) Raji and cured tumours showed very large actively proliferating tumours (data not shown).

In view of the aggressiveness of the cured tumours and their resistance to reovirus *in vitro*, we next assessed the susceptibility of these cells to reovirus treatment *in vivo*. To this end, parental and cured Raji lymphoma tumours grown subcutaneously in SCID mice were subjected to a single intratumoral injection of 1 × 10^7^ PFUs of reovirus. We were surprised to find that, contrary to their behaviour *in vitro*, Raji cured cells were now highly susceptible to reovirus *in vivo* (Figure 4A). To determine if cell death occurred by active viral infection, immunohistochemical (IH) staining with anti-reovirus antibody was performed on paraffin-embedded sections collected 24 days after reovirus injection (Figure 4B). Haematoxylin/eosin staining confirmed the killing of tumour cells in the live virus-treated group, and IH staining with anti-reovirus antibody showed the presence of viral proteins in the residual tumour cells of both Raji parental and Raji cured cells. Therefore, cured cells that are resistant to reovirus *in vitro* become susceptible to reovirus *in vivo*.

### Cured cells are susceptible to *in vitro* infection by protease-stripped virions

Based on current information on reovirus infection strategy, we speculated that the discrepancy between the *in vitro* and *in vivo* data could be due to the proteolytic microenvironment within tumours that enhanced reovirus infection of the cured tumours by converting reovirions into ISVPs. Such ISVPs have been shown to be capable of infecting many restrictive cells that have somehow lost their ability to process the uncoating of the incoming virion (Dermody *et al*, 1993; Baer and Dermody, 1997; Wetzel *et al*, 1997; Baer *et al*, 1999; Golden *et al*, 2002).

**Figure 5:**
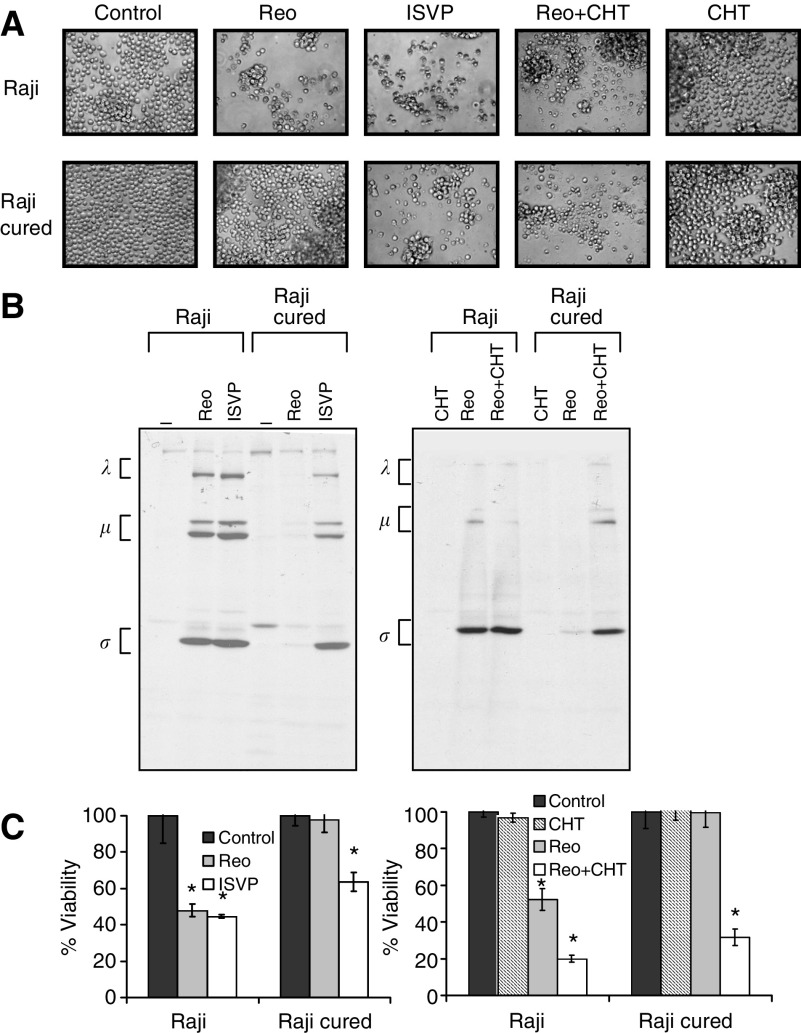
Infection of Raji cured cells by ISVP, or by reovirions in the presence of chymotrypsin. (**A**) Cytopathic effects. Raji parental or cured cells were exposed to ISVP, reovirus or reovirus in the presence of chymotrypsin (CHT) at an MOI of 20 PFU cell^−1^, and photomicrographs were taken at 72 h postinfection. Original magnification × 400. (**B**) Reovirus protein synthesis in infected Raji parental and cured cells. Cells were infected with ISVP, reovirus or reovirus in the presence of CHT, and pulse-labelled with [^35^S]methionine for 6 h at 18 h postinfection. The cells were then harvested and lysed, and reovirus proteins were immunoprecipitated from an aliquot of the lysate using a rabbit polyclonal anti-reovirus antibody, followed by SDS–PAGE. The three size classes of reovirus proteins (*λ*, *μ* and *σ*) are indicated on the left. (**C**) Viability of Raji parental and cured cells infected with ISVP, reovirus or reovirus in the presence of CHT. WST-1 assay was carried out on cells from three independent wells at 96 h postinfection. *P*-values compared with respective uninfected controls are: Left panel, *P*=0.012 (Raji+Reo), *P*=0.017 (Raji+ISVP), *P*=0.61 (Raji cured+Reo) and *P*=0.026 (Raji cured+ISVP). Right panel, *P*=0.019 (Raji+Reo), *P*<0.001 (Raji+Reo+CHT), *P*=0.98 (Raji cured+Reo) and *P*=0.0013 (Raji cured+Reo+CHT). ^*^Indicates statistical significance.

To test this idea, we first determined if resistance of the cured cells to reovirus killing *in vitro* could be reversed by the inclusion of a protease during infection with intact reovirions or by using protease-stripped virions (ISVPs) directly. The results (Figure 5A) show that there was a significant increase in cell death when infection by reovirions was carried out in the presence of chymotrypsin, or when ISVPs were used instead of reovirions. Infected cells were also labelled with [^35^S]methionine, followed by immunoprecipitation with anti-reovirus antibody for the detection of viral protein synthesis (Figure 5B). A drastic increase in synthesis of the three viral protein groups *λ*, *μ* and *σ*, was evident in the Raji cured cells following infection with reovirions in the presence of chymotrypsin or with ISVPs alone. WST-1 assay confirmed the loss in viability of the cured cells from these treatments (Figure 5C). These results clearly show that the resistance of the cured cells to infection with reovirus can be overcome by proteolytic processing of reovirus into ISVP.

### Protease inhibitor E64 prevents reovirus oncolysis *in vitro* and *in vivo*

**Figure 6:**
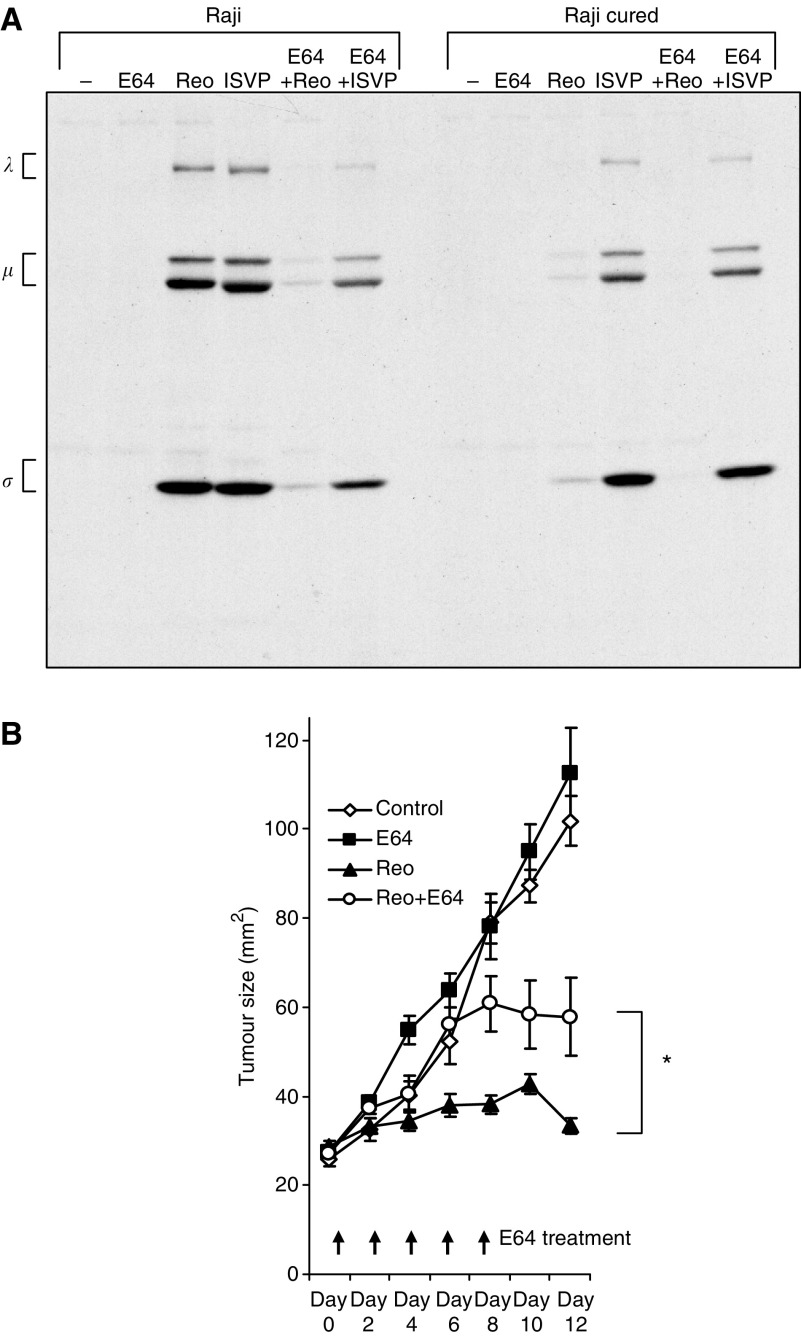
Infection of Raji and Raji cured cells in the presence of E64. (**A**) Reovirus protein synthesis in infected Raji parental and cured cells treated with E64. Cells were infected with ISVP or reovirus in the presence or absence of E64, and pulse-labelled with [^35^S]methionine for 6 h at 18 h postinfection. The cells were then harvested and lysed, and reovirus proteins were immunoprecipitated from an aliquot of the lysate using a rabbit polyclonal anti-reovirus antibody, followed by SDS–PAGE. The three size classes of reovirus proteins (*λ*, *μ* and *σ*) are indicated on the left. (**B**) Intratumoral reovirus therapy of lymphoid tumours in SCID mice exposed to E64 injected i.p. SCID mice were subcutaneously implanted with 1 × 10^7^ cells of Raji parental cells. Following palpable tumour establishment, the tumours received (on day 0) either 1 mg per mouse of E64 in PBS alone (*n*=5) or 1 h before a single intratumoral injection of 1 × 10^7^ PFUs of live reovirus (*n*=5) and E64 was re-injected subsequently every second day until day 8. E64 treatments were compared to PBS control and reovirus alone group (*n*=5). Tumour growth was followed for a period of 12 days and measured two-dimensionally with a caliper. ^*^Wilcoxon's signed-rank test (*P*=0.0379) comparing tumour sizes of reovirus+E64-treated group to reovirus-alone group.

To see if a similar protease processing of virions occurs within Raji cured tumours, which results in their susceptibility to killing by reovirions *in vivo*, we used the specific cysteine protease inhibitor E64, which has been shown to block the conversion of reovirions to ISVPs (Baer and Dermody, 1997; Chandran and Nibert, 1998). We first tested the effect of E64 on Raji and Raji cured cells *in vitro*. The results (Figure 6A) show that E64 effectively blocked infection of the Raji cells by reovirus but not by ISVPs. Raji cured cells were once again only efficiently infected by ISVP and in the presence of E64 as well.

If indeed a proteolytic environment in tumours is important for reovirus permissiveness *in vivo*, as it is for cells *in vitro*, treating mice bearing tumours with a protease inhibitor should also impair reovirus oncolysis *in vivo*. To test this, mice bearing subcutaneous Raji tumours were injected intraperitoneally (i.p.) with E64 before a single intratumoral reovirus injection. I.p. administration of E64 was continued every 2 days until day 8. As observed above, reovirus treatment alone effectively prevented tumour progression; however, reovirus therapy was rendered ineffective in E64-treated mice (Figure 6B). Furthermore, upon termination of the E64 treatment (at day 8), the tumours were seen to regress. These results suggest that the microenvironment inside the cured tumours is proteolytic, and such a proteolytic environment promotes the conversion of reovirions to ISVPs, which can then infect and kill the cured tumour cells that have been selected for their resistance to infection by intact virions.

## DISCUSSION

Resistance to reovirus oncolysis is a phenomenon previously only known to occur *in vitro* for a variety of cell types. This occurs through the acquisition of a PI or cured cellular state (Taber *et al*, 1976; Ahmed *et al*, 1981; Ahmed and Fields, 1982; Danis *et al*, 1993; Dermody *et al*, 1993, 1995; Baer and Dermody, 1997; Wetzel *et al*, 1997; Chappell *et al*, 1998; Baer *et al*, 1999; Ebert *et al*, 2004). The aim of this study was to evaluate the potential limitations that the emergence of PI or cured cells may pose for reovirus oncolytic therapy. To assess this, we investigated the tumorigenicity and subsequent susceptibility to reovirus of a PI Burkitt lymphoma cell line (Raji) and its respective cured and parental counterparts. Our data suggest that these two cellular states are not likely to be a significant barrier to reovirus oncolysis clinically for two reasons: (1) PI Raji cells were not tumorigenic in SCID mice, and (2) while cured Raji cells readily form tumours, they were efficiently eliminated by a single injection of reovirus despite their complete resistance to oncolysis *in vitro*. This latter observation indicates that reovirus' oncolytic properties may be enhanced *in vivo* and that *in vitro* testing of cell lines may not predict *in vivo* susceptibility.

Reovirus is known to produce a persistent infection in a wide range of cell lines including cancer cells (Ahmed *et al*, 1981; Ahmed and Fields, 1982; Dermody *et al*, 1993; Dermody, 1998). Despite this well-described cellular state, this is the first report examining the tumorigenicity of reovirus PI or cured cancer cells. Our present observation that reovirus PI cells regain their tumorigenicity upon clearance of the virus suggests that these PI cells have never lost their intrinsic tumorigenic potential. Rather, this potential is somehow suppressed by the presence of the resident virus. In this regard, it is interesting to note that reovirus PI 3T3 cells reportedly exhibited a decrease of 70–90% in the number of the epidermal growth factor (EGF) receptors and a corresponding decrease in EGF-stimulated DNA synthesis (Verdin *et al*, 1986). Similarly, we observed a marked reduction of activated Ras-GTP and phosphorylated ERK1/2 kinases in the PI cells when compared to parental and cured cells. Reduced expression or activation of important oncogenes and/or growth factors due to the chronic presence of viral proteins and/or transcripts may explain why these cells fail to colonise in soft agar and to form tumours in mice. It is also noteworthy that persistent infection of cancer lines by other oncolytic viruses, such as vesicular stomatis virus (VSV), measles, adenovirus or herpesvirus, also occurs (Minato *et al*, 1979; Wolfson *et al*, 1991; Flomenberg *et al*, 1996; Kao *et al*, 1999), and that measles and VSV PI cells were reportedly incapable of forming tumours *in vivo* (Minato *et al*, 1979; Schattner *et al*, 1985). Similar to the reovirus PI cells reported by Verdin *et al*, measles PI neuroblastoma cells showed a markedly depressed level of H-Ras expression, which correlated with the loss of malignant phenotypes (Wolfson *et al*, 1991). Whether these cells can regain their tumorigenicity upon viral clearance is unknown at present.

Our studies on the Raji cured cells have led us to suggest that proteases present within these tumours are responsible for the enhanced reovirus infection *in vivo*, since these cells are only efficiently infected *in vitro* when reovirus is first proteolytically processed into ISVP before infection. Indeed, addition of exogenous proteases to the culture medium has been shown to facilitate reovirus infection of a variety of cells, which would not otherwise be susceptible (Golden *et al*, 2002). Studies have shown that protease-generated ISVPs are capable of directly penetrating the cell membrane and entering the cytoplasm where viral replication can take place (Borsa *et al*, 1979; Chandran *et al*, 2002). Such a scenario probably plays out in the human gastrointestinal tract where the presence of intestinal proteases likely promotes reovirus infection. It is noteworthy that proteolytic conversion of reovirions into ISVPs in the intestinal lumen has been shown to be essential for efficient reovirus infection in mice (Amerongen *et al*, 1994), and inhibitors of luminal serine proteases reduce viral infectivity (Bass *et al*, 1990). Inhibitors of endosomal and lysosomal proteases have been shown to restrict reovirus infection in cell culture (Dermody *et al*, 1993; Baer and Dermody, 1997; Wetzel *et al*, 1997; Chandran and Nibert, 1998; Baer *et al*, 1999). In this study, we observed that addition of the protease inhibitor E64 blocks reovirus oncolysis of Raji cells *in vitro* but importantly also impairs Raji tumour regression *in vivo*, suggesting the importance of proteases in reovirus oncolysis. Since high levels of protease activity are known to exist in the microenvironment of many tumours, particularly at the tumour–stroma interface, and increased protease production occurs in a variety of transformed cells (Rubin, 2003), tumours present an ideal environment for reovirus replication. Furthermore, cells transformed with oncogenic Ras have increased expression of proteases (Chambers *et al*, 1992), and we have shown that reovirus selectively infects cells with an activated Ras pathway (Coffey *et al*, 1998; Strong *et al*, 1998). Collectively, our data suggest that the proteolytic environment in tumours probably facilitates reovirus uncoating, and this could account for its enhanced oncolytic capability *in vivo*.

In view of the reovirus clinical trials currently underway, results from this study should alleviate some concerns regarding the potential problems with the tumorigenic potential of PI tumour cells, as well as with the resistance of the cured tumour cells to reovirus therapy. These results further suggest that reovirus could have broadened oncolytic capabilities due to enhanced proteolytic processing of virions within tumours, and that *in vitro* testing of cancer cells or tumour specimens does not necessarily predict which tumours will be resistant to reovirus infection *in vivo*.
